# Peer Review of “Effects of Pharmacogenomic Testing in Clinical Pain Management: Retrospective Study”

**DOI:** 10.2196/37145

**Published:** 2022-05-03

**Authors:** Komal Rathi

**Affiliations:** 1 The Children's Hospital of Philadelphia Philadelphia, PA United States

**Keywords:** pharmacogenomics, pain management, drug-drug interaction, DDI, pharmacy, prescriptions, genetics, genomics, drug-gene interaction, pain

*This is a peer-review report submitted for the paper “Effects of Pharmacogenomic Testing in Clinical Pain Management: Retrospective Study.”*

## Review Round 1

### General Comments

This paper [1] touches a very important and clinically relevant issue of adverse drug interactions with genetic variations and how these variants affect the patient’s response to the specific drug. It focuses on utilizing pharmacogenetic (PGx) testing in clinical practice, which takes into account these relevant drug-genome interactions when prescribing drug therapy. They appropriately chose an acceptable sample size >150 and follow them for a significant period of time (>18 months). Importantly, they have performed retrospective studies, which makes a good case for the utility of PGx testing. They also lay a good background on what other technologies for PGx testing are being routinely used in current clinical settings.

### Specific Comments

#### Major Comments

I have no negative comments for this paper, here are some positive comments:

1. I especially find it very impressive that various figures and tables were added to the paper, which shows their thorough work. Figure 1 clearly describes PGx testing compared to urine drug toxicology reports. Figure 2 indicates the potential drug-gene and drug-drug interactions as provided by the PGx testing and suggests alternatives in case of serious and moderate interactions based on information from various regulatory bodies. Tables 1 and 2 are of significant interest because they focus on genotype, phenotype, and population frequencies for the genes in the panel. Figure 3 focuses on the importance of PGx testing in identifying moderate to serious drug-drug or drug-gene interactions.

Overall, I find this study very impactful especially with the advent of individualized drug therapy and targeted drug recommendations.

2. The results and discussion focus on how recommendations and dosage were changed based on PGx reports and resulted in favorable outcomes for the patients. This shows the utility of PGx in areas where health care professionals are not aware of these interferences or interactions between drug-gene and drug-drug.

3. I am not sure how many clinically relevant genes have changed or updated since April 2016, but this paper lays the groundwork for a more up-to-date gene panel to be used. I would be interested in seeing the outcome with a more up-to-date gene list but that does not necessarily have to be addressed in this paper.

#### Minor Comments

4. This was a very legibly worded paper, and I found no issues with the English or the scientific language that was used.

## Abbreviations

PGx: pharmacogenetic
